# The Role of Medial Plantar Artery Flap in Heel Pad Reconstruction: A Report of Two Cases

**DOI:** 10.7759/cureus.41844

**Published:** 2023-07-13

**Authors:** Zhi Sing Oon, Ren Yi Kow, Akmal Azim Ahmad Alwi, Mohd Adham Shah Ayeop, Chooi Leng Low, Aminudin Che Ahmad

**Affiliations:** 1 Department of Orthopedics, Queen Elizabeth Hospital, Kota Kinabalu, MYS; 2 Department of Orthopedics, Traumatology, and Rehabilitation, International Islamic University Malaysia, Kuantan, MYS; 3 Department of Plastic and Reconstructive Surgery, International Islamic University Malaysia, Kuantan, MYS; 4 Department of Radiology, International Islamic University Malaysia, Kuantan, MYS

**Keywords:** diabetes mellitus, diabetic foot management, heel flaps, heel reconstruction, heel pad, medial plantar flap, medial plantar artery perforator flap, medial plantar artery flap

## Abstract

The heel and sole possess unique anatomical characteristics that serve a weight-bearing and shock-absorbing function. The heel is particularly vital, as any defects in this area can lead to gait instability. Reconstructing a heel defect presents challenges, as the donor flap must be durable enough to withstand high force loads while also providing protective sensation. Recently, the medial plantar artery flap has been successfully employed for the reconstruction of defective heel pads. This flap offers glabrous skin capable of weight transmission and intact protective sensation. In this report, we present two cases of heel pad loss secondary to chronic diabetic complications and trauma, respectively, which were treated with medial plantar artery flap reconstruction. Both cases were successfully treated, and they showed a good functional outcome.

## Introduction

The foot has been crucial in enabling essential actions such as walking, running, and jumping ever since humans developed an evolutionary adaptation for a bipedal gait [1-5]. The heel and sole have thick glabrous skin capable of weight transmission and providing protective sensation during a normal gait [6]. Soft tissue defects in the heel region can result from trauma, burns, infection, necrosis, chronic ulcers, and tumor resection [7]. The ideal reconstruction of the heel pad requires the use of soft tissue that can withstand body weight and provide sensation to prevent pressure injuries. The skin of the instep area, based on the medial plantar artery, is often selected for reconstructing medial and plantar heel defects due to its ability to meet these demands [7,8]. In this report, we present our experience in managing two patients with heel pad loss caused by diabetic complications and trauma, respectively, who were successfully treated with reconstruction using the medial plantar artery flap. We also discuss the functional outcomes of these treatments.

## Case presentation

Case 1

**Figure 1:**
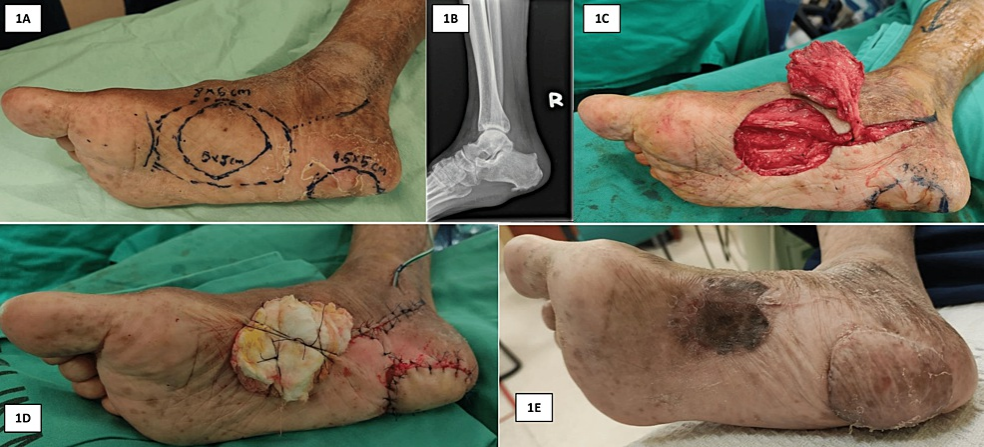
A: The patient presented with a chronic ulcer at the right heel with an absent heel pad. B: Plain radiograph of the ankle revealed minimal soft tissue shadow overlying the calcaneum, and there was no abnormality in the calcaneum. C: Medial plantar artery flap was raised. D: The flap was anchored to the heel, and the medial plantar area was covered with a full-thickness skin graft. E: The patient recovered well with no complications.

A 79-year-old male with a history of diabetes mellitus of at least two decades presented with a chronic ulcer at the right heel with an absent heel pad (Figure 1A). He had undergone previous debridement of the affected heel due to an infected diabetic foot ulcer, resulting in the loss of the heel pad. Examination of the foot revealed an absent heel pad with a thin, ulcerated skin over the calcaneus. The remaining plantar skin appeared healthy. Vascular examination indicated palpable posterior tibial and dorsalis pedis arteries with good blood flow. Radiography of the right ankle revealed minimal soft tissue shadow overlying the calcaneum, and there was no abnormality in the calcaneum (Figure 1B). The patient was offered a soft tissue reconstruction with a medial plantar artery flap.

The patient underwent reconstructive surgery in a supine position under general anesthesia. Tourniquet was used during the surgery. Prior to the surgery, a sterile handheld Doppler ultrasound was utilized to determine the course of the perforator branches of the medial plantar artery. The skin paddle was designed 6 cm in length and 5 cm in width and was marked. Flap dissection commenced with an incision along the distal border of the flap down to the fascia, which was then dissected along a plane superficial to the abductor hallucis muscle (Figure 1C). The distal branches of the medial plantar artery were identified and divided. The neurovascular bundles were raised in continuity with the flap. The abductor hallucis muscle was divided proximally to facilitate the mobilization of the pedicle, and it was reapproximated with an absorbable suture. The plantar skin defect at the heel was excised, and the flap was sutured into the heel defect (Figure 1D). A full-thickness skin graft was harvested from the contralateral groin and secured to the donor site. Once the tourniquet was released, immediate vascularization of the flap was observed. Six weeks after surgery, the healing of the flap was promising, and the patient was allowed partial weight-bearing. Six months following surgery, the patient was able to fully weight bear on the foot. The patient did not experience any complications such as heel pain or ulceration (Figure 1E).

Case 2

**Figure 2:**
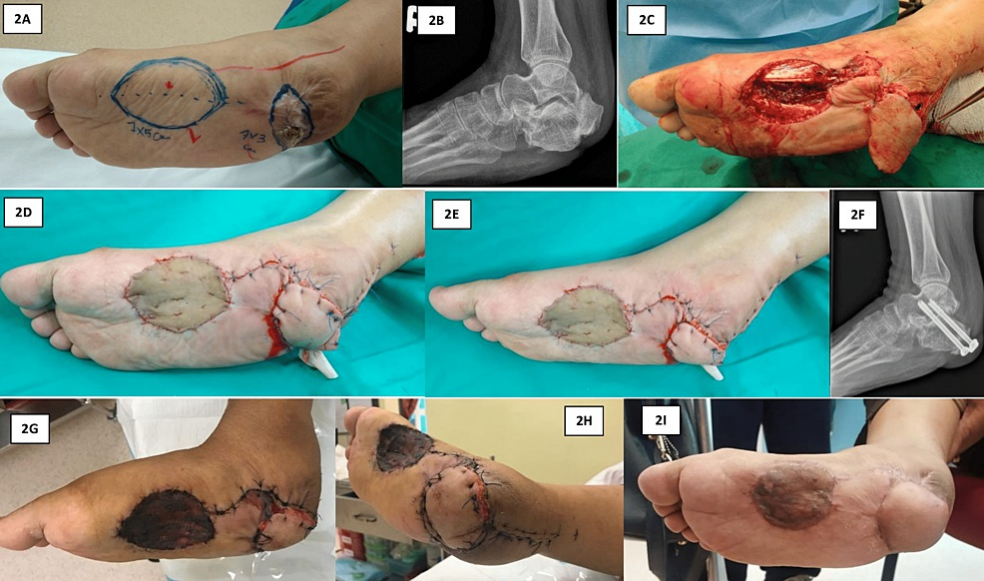
A: The patient presented with right foot equinus deformity and loss of heel pad with skin contracture. B: Plain radiograph of the ankle revealed a malunion of the calcaneum with incongruence of the subtalar joint. C: The medial plantar artery flap was raised intraoperatively. D and E: The medial plantar artery flap was anchored to the heel, and the donor site was covered with a full-thickness skin graft. F: Plain radiograph of the ankle revealed a successful subtalar fusion. G and H: The healing of the flap and skin graft was satisfactory six weeks after the surgery. I: The patient recovered fully with a near-normal range of movement of the ankle.

A 46-year-old female with no known comorbidities suffered an open fracture of the calcaneum with heel pad loss due to a frictional injury to the heel. She presented to us with a painful heel and difficulty walking. She was using an assistive device and walking in a tiptoeing gait. Physical examination revealed a right foot with equinus deformity and an absent heel pad with skin contracture (Figure 2A). Palpation of the dorsalis pedis and posterior tibial pulses showed good volume. Radiography revealed malunion of the calcaneum and incongruence of the subtalar joint (Figure 2B). Due to her high functional demand and the need to reconstruct the calcaneum height, prevent subtalar arthritis, and provide a functional heel, we offered a treatment plan consisting of subtalar fusion, tendon Achilles lengthening, and soft tissue reconstruction using a medial plantar artery flap.

Intraoperatively, the patient was positioned supine under general anesthesia. A thigh tourniquet was utilized during the surgery. Mapping of the course of the medial plantar artery and its perforator branch was performed prior to the surgery. The skin paddle was measured and identified. The malunion calcaneus was addressed first during the surgery where an osteotomy and bone grafting were performed to reconstruct the calcaneal height. Subsequently, subtalar arthrodesis was carried out using cannulated screws. A Z-plasty lengthening of the Achilles tendon was performed to correct the equinus deformity.

Following that, a skin incision was made over the medial plantar surface. The incision deepened, and dissection was done until the plane was superficial to the abductor hallucis brevis. Vascular pedicles were identified and raised with the flap. The flap was mobilized posteriorly to cover the heel defect (Figure 2C). A full-thickness skin graft was harvested from the right groin and anchored to the medial plantar surface (Figure 2D and Figure 2E).

Six weeks following the surgery, the plain radiograph of the ankle demonstrated a successful subtalar fusion (Figure 2F). The patient was allowed partial weight-bearing, and the healing process of the flap and skin graft was satisfactory with no evidence of infection or ulceration (Figure 2G and Figure 2H). The range of motion of the ankle was up to 15 degrees of dorsiflexion. One year after the surgery, the patient was able to fully bear weight and regain near-normal dorsiflexion. The patient was very satisfied with the outcome of the surgery (Figure 2I).

## Discussion

The skin covering both the heel and sole of the foot has an intricate connective tissue system. It is a highly specialized tissue structure containing thick glabrous skin, thick epidermis and dermis, fibroadipose tissue, and abundant fibrous septae connecting the skin to the plantar aponeurosis [9]. Reconstruction of this specialized area remains a challenging task due to the scarcity of glabrous local tissue. Various surgical options are available for the reconstruction of the heel, each with its respective advantages and disadvantages. These options include skin grafts, local advancement flaps, island pedicle flaps, locoregional flaps, cross-leg flaps, and free flaps, among others [10,11].

The aim of reconstruction surgery is to recreate a heel as close to anatomical contour as possible, using durable, sensible, glabrous, and stable tissue, thereby reproducing a functional heel [12]. The only source of such tissue is the instep flap of the foot. The entire instep skin can be raised as a fasciocutaneous flap based on the medial plantar vessels [13]. The medial plantar artery flap was originally described by Harrison and Morgan in 1981 [14]. The posterior tibial artery divides into the larger lateral plantar artery and the smaller medial plantar artery. The medial plantar artery initially courses deep into the abductor hallucis muscle and then exits distally as it runs between the intermuscular septum and flexor digitorum brevis muscle, terminating by anastomosing with the first plantar metatarsal artery. The medial plantar nerve runs laterally to the artery along its course. The medial plantar artery flap has several advantages, including similar skin anatomy to that of the surrounding plantar skin, a constant blood supply, and a cutaneous nerve supply for protective and tactile sensation [15]. The drawback of this instep flap is its limitation in size; hence, the coverage of deep and extensive defects requires a larger muscle or fasciocutaneous flap [15].

To facilitate the selection of appropriate reconstructive flaps in clinical applications, researchers have proposed various principles. Krishna et al. [11] proposed a treatment algorithm for heel soft tissue based on the location of the defect, whether it is an anterior defect (weight-bearing part), posterior defect (non-weight-bearing part), or complete defect. For instance, in a moderate-sized anterior defect with an intact medial plantar artery, it is best to utilize a medial plantar artery flap to cover the defect. Feng et al. [16] proposed the “five-zone method,” which comprises the inner heel, the heel plantar, the posterior heel, the outer heel, and the articular zone. Their research concluded that the medial plantar island flap is suitable for small (<5 cm) defects localized in the medial, posterior, and plantar areas of the heel. In our cases, both of our patients have had a heel defect in the weight-bearing area, and the size of the defect is within 5 cm; hence, a medial plantar artery flap was a suitable reconstructive option.

One of the complications of heel pad reconstruction at the recipient site is ulceration. The reported incidence rate of ulceration ranges from 33% to 42.9% among all heel pad reconstructions [17]. The factors affecting the occurrence of ulceration were found to be continuous pressure by bony prominences, flap inset, and improper footwear [14]. Bhandari and Srivastava [18] conducted a study involving 29 patients with soft tissue defects treated with a medial plantar artery flap, and no ulceration developed in 11 patients who followed up for 22 years. Other reported minor flap complications included partial flap necrosis, delayed flap healing, infection, and hematoma [19]. With this in mind, we have advised our patients for non-weight-bearing at the first six weeks postoperatively, followed by partial weight-bearing after six weeks. This measure ensures the healing process of the flap and prevents possible ulcerations. Both of our patients did not develop any ulceration one year after the surgery. The flaps did not become atrophy one year after the surgery. Nevertheless, a longer duration of follow-up is required.

## Conclusions

A highly specialized soft tissue is required for the reconstruction of the heel pad. The soft tissue needs to contain thick glabrous skin, thick epidermis, and dermis while providing protective and tactile sensation. In this report, two patients with heel pad loss caused by diabetic complications and trauma, respectively, were successfully treated with reconstruction using the medial plantar artery flap. The medial plantar artery flap is a suitable option for the reconstruction of soft tissue defects in the weight-bearing portion of the heel, resulting in excellent functional outcomes.
